# Effects of caffeine and acute exercise on the validity of a submaximal cycle ergometer test: a randomised, placebo-controlled crossover study

**DOI:** 10.1007/s00421-026-06182-0

**Published:** 2026-02-28

**Authors:** Frida Björkman, Jessica Engström, Torbjörn Helge, Örjan Ekblom

**Affiliations:** 1 Department of Physical Activity and Health, The Swedish School of Sport and Health Sciences, Stockholm, Sweden; 2 Department of Physiology, Nutrition and Biomechanics, The Swedish School of Sport and Health Sciences, Stockholm, Sweden

**Keywords:** Submaximal exercise testing, VO_2_max estimation, test validity, caffeine, Ekblom-Bak test

## Abstract

**Purpose:**

To investigate the effects of caffeine (Caf) consumption and prior exercise on the validity of the Ekblom–Bak cycle test (EB-test) for estimating maximal oxygen uptake (VO₂max).

**Methods:**

Healthy, habitual caffeine users received 250 mg Caf or placebo (Pla) in a double-blind randomised crossover design separated by one week. Each visit included two EB-tests performed before and after a maximal treadmill exercise (Ex). Heart rate (HR), blood pressure (BP), and oxygen uptake were measured, and estimation error was calculated as estimated–measured VO₂max. Data were analysed using repeated measures ANOVA with condition (Caf, Pla) and time (pre-Ex, post-Ex) as within-subject factors.

**Results:**

Participants were 15 women and 17 men (33.5 ± 11.1 years). Resting HR and BP did not differ between trials, except for a modest increase in systolic BP after Caf (129 ± 14 mmHg vs. 122 ± 15 mmHg; *p* = 0.042). Maximal HR and VO₂max were unaffected, but time to exhaustion increased with caffeine (438 ± 73 s vs. 415 ± 69 s; *p* = 0.012). HR increased significantly from pre- to post-Ex, with no differences between Caf and Pla conditions. Estimation errors rose from pre- to post-Ex (–0.17 and − 0.12 L·min⁻¹ to − 0.28 and − 0.30 L·min⁻¹), independent of caffeine intake, body mass, or measured VO₂max.

**Conclusion:**

Caffeine did not affect the accuracy of the VO₂max estimation from the EB-test. However, strenuous exercise before testing increased estimation errors, suggesting that recent intense exercise has a greater impact on EB-test validity than moderate caffeine consumption.

## Introduction

Maximal oxygen uptake (VO_2_max) is a good measure of cardiorespiratory fitness and a strong predictor of overall health (Blair et al. 1989). Direct measurement of VO₂max is not always feasible due to time constraints, the need for specialised equipment and trained personnel, and the requirement for maximal exertion that may not be appropriate for all participants. Therefore, submaximal exercise tests are often used to estimate VO₂max (Astrand and Ryhming 1954; Noonan and Dean 2000). The Ekblom-Bak test (EB-test) is a heart rate (HR) based submaximal cycle test that has demonstrated good validity across ages and fitness levels, with a coefficient of variation of ~ 9% and a standard error of the estimate of ~ 0.25 L min^− 1^ (Björkman et al. 2016; Ekblom-Bak et al. 2014).

Caffeine is a widely used stimulant found in coffee, tea, energy drinks, and supplements, and caffeinated beverages are regularly consumed by approximately 90% of adults worldwide (Čižmárová et al. 2025). Its physiological effects stem from stimulation of the nervous system, mainly through adenosine receptor blockade (Schwabe et al. 1985) and increased activity of the autonomic nervous system, resulting in the release of plasma catecholamines, with dose-dependent increases in blood pressure (BP) (Abbas-Hashemi et al. 2023). The performance-enhancing effects of caffeine during physical exercise include delayed time to exhaustion and lower ratings of perceived exertion (RPE) (Doherty and Smith 2004; Pereira et al. 2016). Although findings on the effects of caffeine on VO₂max are conflicting, probably due to differences in participant characteristics, habitual caffeine consumption, dosing strategies, and exercise protocols, the overall evidence suggests that caffeine has no significant effect on VO₂max (Marinho et al. 2024). Instead, caffeine’s ergogenic effects are primarily observed in performance-related outcomes (e.g., time to exhaustion, power output, or perceived exertion), rather than maximal oxygen uptake per se. Ergogenic effects are achieved at doses > 2 mg·kg⁻¹ of body mass, whereas the optimal dose is 3–6 mg·kg⁻¹ (Guest et al. 2021). Higher doses are not associated with further improvements in performance but may cause side effects such as gastrointestinal discomfort, headache and tachycardia (de Souza et al. 2022; Guest et al. 2021).

Caffeine can alter HR responses across various exercise intensities (Bell et al. 1998; McNaughton 1987; Sung et al. 1995), but the results are inconclusive. Some studies have found that caffeine doses ranging from 1.5 to 5.0 mg·kg⁻¹ of body weight lowered HR during a wide range of submaximal work rates (Gaesser and Rich 1985; Glaister et al. 2021; McClaran and Wetter 2007; Turley and Gerst 2006), while other studies report increased HR using doses of 3–6 mg·kg⁻¹ in well-trained (Desbrow et al. 2012) and sedentary (Laurence et al. 2012) participants, respectively. Other studies have not found any caffeine-induced change in HR response during exercise (Bangsbo et al. 1992; Daniels et al. 1998; Engels et al. 1999; Flinn et al. 1990; Tarnopolsky et al. 1989). A recent meta-analysis examined the effects of ordinary to semi-high doses of caffeine and found no significant impact on HR response during work rates corresponding to 60–85% of VO_2_max (Glaister and Gissane 2018). Furthermore, habitual coffee consumption of 3 to 6 cups per day appears not to affect resting HR (Han et al. 2023). The differences among studies may be partly explained by variations in caffeine dose, timing, and administration, as well as differences in exercise intensity, duration, and participants’ habitual caffeine use. Taken together, caffeine’s effects on HR during exercise appear inconsistent, whereas its ergogenic effects on endurance and high-intensity exercise performance are more consistently reported. In the context of the present study, the administered caffeine dose falls within this ergogenic range. Furthermore, any caffeine-related effects are more likely to influence cardiovascular responses or exercise tolerance rather than VO_2_max per se.

The accuracy of VO₂max estimates from submaximal tests depends on several factors, including standardisation, medication, caffeine intake, and prior physical activity. For instance, the EB-test has been shown to systematically overestimate VO₂max in individuals using beta blockers (Östh et al. 2025). Although caffeine can affect exercise test results (Doherty and Smith 2004), its impact on HR–based submaximal tests remains unclear. Similarly, it is unknown how recent strenuous exercise affects the validity of these predictions. Strenuous aerobic exercise places the cardiovascular system in a distinct recovery state, characterised by postexercise hypotension and sustained vasodilation, mediated by central autonomic adjustments and local vascular mechanisms, along with reduced cardiac preload, fluid shifts, and thermoregulatory demands (Romero et al. 2017). Because HR-based submaximal tests, such as the Ekblom-Bak test, assume a stable HR–work rate relationship, performing them during this physiologically altered recovery state may introduce systematic errors in VO₂max estimation.

Given these uncertainties, further research is necessary to clarify the impact of caffeine and prior exercise on the validity of submaximal exercise tests. The aim of the present study was to examine the effects of caffeine intake on HR responses and the estimated VO₂max derived from the EB-test. A secondary aim was to investigate how strenuous exercise prior to the test affected the aforementioned variables. It was hypothesised that estimation error would be higher after caffeine ingestion and after prior exercise compared with placebo and rested conditions, with each factor expected to independently contribute to the increase in error. The specific research questions were:

1. Does the validity of the EB-test change after ingestion of 250 mg of caffeine?
2. Does the validity of the EB-test change after performing strenuous exercise prior to testing?
3. Are any of the changes in validity (from questions 1 and 2) associated with body mass or cardiorespiratory fitness level (VO₂max)?

## Method

### Study participants

Study participants were recruited through public advertisements. Eligible individuals were men and women aged 18–55 years, non-smokers, free from illnesses, injuries, or disabilities affecting physical performance or caffeine metabolism, and with current experience of caffeine consumption (e.g., self-reported habitual intake of coffee, tea, energy drinks, or caffeine tablets). Individuals taking medications known to affect HR or interact with caffeine, or those with hypersensitivity to caffeine, were excluded. Additional contraindications were assessed via a health declaration.

All participants received written and verbal information about the study procedures, potential risks and discomforts, and their right to withdraw at any point. Written informed consent was obtained. The study adhered to the Declaration of Helsinki and was approved by the Regional Ethics Committee in Stockholm (reference number 2023-03730-01). Preregistration was completed on October 27, 2023, on the Open Science Framework (osf.io), DOI: 10.17605/OSF.IO/GU5YP.

### Sample size determination

This study employed a randomised crossover design in which all participants received each experimental treatment. Based on previous validation studies of the Ekblom–Bak (EB) test, the mean VO₂max in the relevant age group was 3.63 ± 1.05 L·min⁻¹, with a test–retest coefficient of variation of 6.2% (Björkman et al. 2016; Ekblom-Bak et al. 2014). The study was designed to detect a minimum change of 3% in VO₂max following caffeine intake or exercise. Using this information, the within-subject SD of differences was estimated as 0.225 L·min⁻¹. An a priori power analysis was performed using G*Power 3.1 (Heinrich Heine University, Düsseldorf, Germany) for a two-tailed paired t-test (α = 0.05, power = 0.80), which indicated that 37 participants were required to detect this effect size (Cohen’s d = 0.48).

### Study design

The study was conducted in a double-blind and placebo-controlled manner with a crossover design. The EB-test was validated against measured VO_2_max, both with and without the influence of caffeine intake, and with and without prior physical exercise. The experimental conditions were caffeine (Caf) and placebo (Pla). Block randomisation was used to prevent severe imbalances in sample allocation. Each participant was randomly assigned to one of the ten equally sized, predetermined blocks. The random number list used to create these four blocks was created using an Excel spreadsheet with the RAND formula, “=RAND()”. A research assistant generated the random allocation sequence. An external researcher prepared identical administration tablets in numbered bags, labelling the two conditions as “A” and “B.” Participants were enrolled by the external researcher and assigned to interventions by a research assistant. Neither the participants nor the research assistant conducting the tests were aware of the allocation, ensuring a double-blind study.

In the weeks preceding the experiments, all participants visited the Laboratory for Applied Sports Science (LTIV) at the Swedish School of Sport and Health Sciences to familiarise themselves with the testing procedures. During this visit, participants completed an informed consent form, a health declaration, a cardiac screening questionnaire, and a brief questionnaire about caffeine habits. Subsequently, each participant performed an individually designed maximal running test to determine VO₂max (characterisation test).

**Fig. 1:**
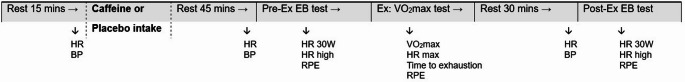
Timeline of experimental procedures. Ex; exercise, EB-test; Ekblom-Bak test, HR; heart rate, BP; blood pressure, RPE; ratings of perceived exertion, VO_2_max; maximal oxygen uptake

The main experiments were conducted on two separate days, one week apart. Before the visit, participants were asked to refrain from caffeine, smoking, and vigorous physical activity for 24 h prior to the test, and not to consume any heavy meals within 3 h of the test. During the test day, participants were allowed to consume water *ad libitum*. All tests were performed in a climate-controlled laboratory environment, with the temperature maintained between 19.0 °C and 19.2 °C. The timeline of measurements for each experimental day is shown in Fig. 1. Body mass (measured while wearing lightweight clothes to the nearest 0.1 kg) and height (to the nearest 0.1 cm) were recorded, and participants were equipped with an HR monitor (Polar Electro, Kempele, Finland). All HR recordings were derived from the HR monitor. BP was measured on the right upper arm with an automatic oscillometric device (Omron M6 Comfort, model HEM-7221-E; Omron Corporation, Kyoto, Japan). The first measurements of HR and BP were conducted after 15 min of supine rest. Following 15 min of supine rest, baseline HR and BP were measured. Participants then ingested either 250 mg of caffeine (condition: Caf) or 50 µg of vitamin D (condition: Pla), administered in tablet form with a MEDCOAT^®^ coating, which facilitates swallowing and masks flavour, and ensured that the tablets were visually identical in size and shape. After ingestion, participants rested for 45 min, after which HR and BP were reassessed.

Each experimental day included two submaximal EB-tests and one maximal treadmill running test to determine VO₂max. The EB-tests were conducted on a mechanically braked cycle ergometer (Monark Model 828E, Varberg, Sweden). After standard adjustment of the seat and handlebar, participants were provided with verbal instructions on the Borg’s RPE scale (Borg 1970) and were instructed to maintain a constant pedalling rate of 60 revolutions per minute throughout the test. The EB-test comprises 4 min of cycling at a standard work rate (0.5 kp, i.e., ~ 30 W), followed by 4 min of cycling at a higher, individually prescribed work rate to achieve a RPE of ≈ 14. The higher work rate was determined by the test leader based on sex, self-reported physical status, and exercise habits. HR was recorded as the average of the final minute at each work rate, and perceived exertion was rated using the RPE scale immediately after completing each work rate. Full details of the test procedure are described elsewhere (Ekblom-Bak et al. 2014). Participants cycled on a mechanically braked cycle ergometer (Monark Model 828E, Varberg, Sweden). The first cycle test (referred to as “EB pre-Ex`”) was performed 45 min after ingestion of caffeine or placebo. After a brief rest (maximum of 5 min), participants performed a strenuous exercise session (Ex) consisting of a graded maximal running test. The running test began with a 5-minute warm-up, during which participants started walking at a speed of 5–6 km/h, gradually increasing to a comfortable running pace. The test protocol was individually adjusted for starting speed and incline but followed a standardised format: the speed or incline was increased every minute until the participant reached voluntary exhaustion. Oxygen uptake was measured during the maximal running test using a Jaeger Oxycon Pro metabolic system (Hoechberg, Germany) with a Hans Rudolph facemask. Gas, volume, and ambient conditions were calibrated before each test. VO₂max was considered valid when a plateau in VO₂ occurred despite increasing speed or incline (Howley et al. 1995), supported by a respiratory exchange ratio ≥ 1.10, RPE > 16, exercise duration > 6 min, and a maximal HR within ± 10–15 beats per minute (bpm) of the age-predicted maximum (Poole and Jones 2017). The maximal test was followed by a 30-minute rest period, after which participants completed a second EB-test (“EB post-Ex”). VO₂max was estimated using validated sex-specific prediction equations that incorporate age and heart rate–work rate relationships derived from the two submaximal exercise stages (Björkman et al. 2016).

### Statistics

The study aimed to manipulate HR responses at two work rates during a submaximal cycle ergometer test by introducing 250 mg caffeine and/or strenuous exercise (Ex) prior to testing. The primary outcome variables in the statistical analyses were the estimation errors, i.e., the differences between the estimated and measured VO_2_max values under the different conditions. The secondary outcomes were resting cardiovascular variables (resting HR, systolic and diastolic BP), the submaximal HR values obtained during the EB test at low (30 W) and high work rates, as well as the exercise performance variables (time to exhaustion, measured VO₂max, and maximal HR) recorded during the experimental trials. Normality of the data was evaluated using the Shapiro–Wilk tests. Descriptive statistics are reported as mean ± standard deviation (SD) for continuous and normally distributed variables. Paired-samples t-tests were used to analyse differences in descriptive variables, with effect sizes reported as Cohen’s d. To account for multiple comparisons, p-values were adjusted using the Bonferroni–Holm correction method.

The multiple parameters from the EB-test and the differences in estimation error between conditions and time points were analysed using a two-way repeated-measures analysis of variance (RM ANOVA) with condition (Pla, Caf) and time (pre-Ex, post-Ex) as within-subject factors. Significant interaction effects were followed up with Tukey’s post hoc pairwise comparisons. In addition, equivalence testing was performed using the two one-sided tests (TOST) procedure. Equivalence bounds were set at ± 0.1 L·min⁻¹ to evaluate whether the estimation errors during Caf and Pla trials, in resting and exercised conditions, were statistically equivalent. For reference, previous validation studies report a typical estimation error of – 0.05 L·min⁻¹ among age-matched subjects (Björkman et al. 2016), and the ± 0.1 L·min⁻¹ bounds were applied to capture changes larger than the typical noise, but not trivially small fluctuations. To account for potential confounding effects of inter-individual differences, additional analyses were conducted using RM analysis of covariance (ANCOVA). Body mass and cardiorespiratory fitness level (i.e., measured VO_2_max) were entered as covariates to control for their potential influence on the estimation error. Although normality of the dependent variables was confirmed through Shapiro–Wilk tests, the normality of ANOVA residuals was also evaluated using Q–Q plots. Visual inspection indicated that residuals were approximately normally distributed. Effect sizes for ANOVA results are reported as partial eta squared (partial η²). Significance was set at *p* < 0.05. All statistical analyses were performed in Jamovi (version 2.6). This study was conducted in accordance with the CONSORT (Consolidated Standards of Reporting Trials) guidelines (Schulz et al. 2010).

### Results

Thirty-two habitual caffeine users volunteered to participate in the study (15 women and 17 men). Four subjects (two women and two men) had to withdraw from the study due to different illnesses and injuries. Of the 28 participants who completed the study, 13 received Caf first, and 15 received Pla first. In this crossover design, all randomised participants who completed all experimental sessions were included in analyses for all outcome variables. No adverse events or unintended effects were reported in any condition. The periods of participant recruitment and follow-up were from October 20, 2023, to March 14, 2024. The trial was completed as scheduled.

Subject characteristics are presented in Table 1. In this study, the administered dose of 250 mg caffeine corresponded to 3.33 ± 0.57 mg·kg⁻¹ body mass for the participants. The resting values of HR and BP are presented in Table 2.

**Table 1:** Subject characteristics

	All (*n* = 28)	Men (*n* = 15)	Women (*n* = 13)	*p* =
Age (years)	33.5 ± 11.1	35.6 ± 11.1	31.1 ± 11.1	0.29
Height (cm)	175 ± 9.0	180.9 ± 6.82	168.5 ± 6.18	< 0.001
Body mass (kg)	77.2 ± 13.5	84.6 ± 11.4	68.7 ± 10.7	< 0.001
VO_2_max (L min^− 1^)*	3.78 ± 0.9	4.43 ± 0.67	3.04 ± 0.33	< 0.001
VO_2_max (ml kg^− 1^ min^− 1^)*	49.1 ± 8.1	52.8 ± 8.1	44.7 ± 5.5	0.005

Values are presented as mean ± SD. The p-values denote differences between men and women. VO_2_max; maximal oxygen uptake

*VO_2_max values from the characterisation test

**Table 2:** Resting values of heart rate and blood pressure

	Pla trial	Caf trial	*p* =	Cohen’s d
HR (bpm) baseline	67 ± 13	64 ± 11	0.436	0.31
HR (bpm), post tablet intake^†^	61 ± 10	57 ± 11	0.096	0.49
HR (bpm), post exercise*	91 ± 14	92 ± 14	0.678	-0.20
SBP (mmHg), baseline	125 ± 15	125 ± 12	0.957	0.01
SBP (mmHg), post tablet intake^†^	122 ± 15	129 ± 14	0.042	-0.56
DBP (mmHg), baseline	79 ± 11	77 ± 8	0.678	0.23
DBP (mmHg), post tablet intake^†^	79 ± 10	83 ± 10	0.195	-0.41

Values are mean and standard deviation (SD). *p*-values adjusted using the Bonferroni–Holm correction for multiple comparisons

*Pla* placebo, *Caf* caffeine, *HR* heart rate, *bpm* beats per minute, *SBP* systolic blood pressure, *DBP* diastolic blood pressure

^*^HR was measured following a 30-minute rest period after the maximal exercise test

^†^HR and BP were measured following a 45-minute rest period after tablet ingestion

No significant differences were observed for measured VO_2_max and maximal HR (3.81 ± 0.94 L min^− 1^ and 187 ± 13 bpm, *p* = 0.93, during the Pla trial and 3.81 ± 0.93 L min^− 1^ and 188 ± 12 bpm, *p* = 0.12, during the Caf trial, respectively). However, there was a significant difference in time to exhaustion: 415 ± 69 s during the Pla trial compared to 438 ± 73 s during the Caf trial (*p* = 0.012), respectively.

**Fig. 2:**
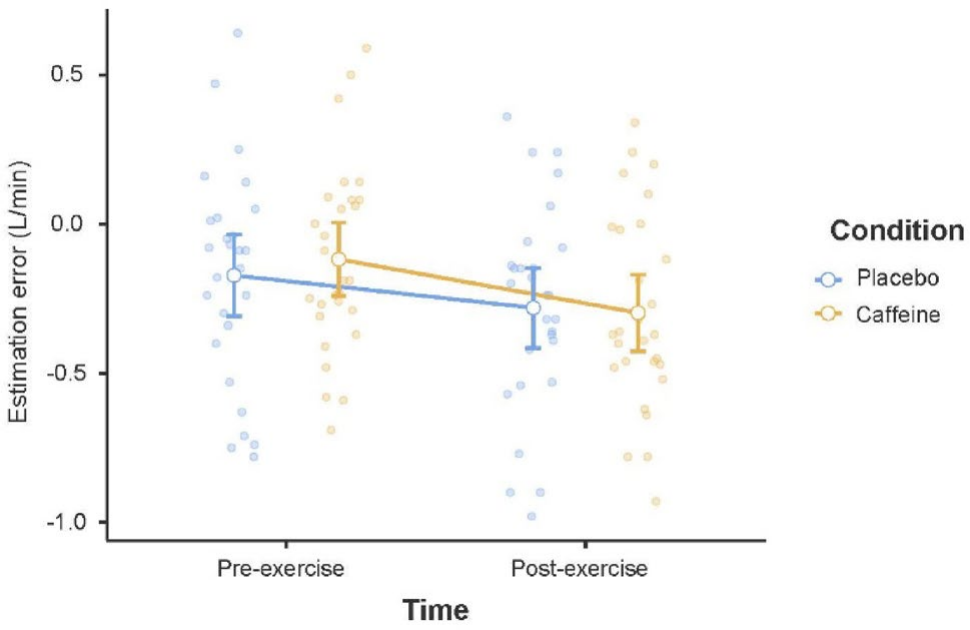
Estimated marginal means of estimation error across conditions (caffeine vs. placebo) and time points (pre- and post-exercise), derived from a repeated-measures ANOVA. Error bars represent 95% confidence intervals

The primary outcome variables (measurement errors), as well as the submaximal HR recordings, Borg scale ratings and estimated VO_2_max values from the EB-test, are reported in Table 3. The unadjusted values from the RM ANOVA revealed a significant time effect, with post-exercise HR values increasing 13–27%, resulting in an approximately 5% greater underestimation of VO_2_max during the second EB-test, regardless of whether participants received caffeine or placebo. A significant time effect was also observed for RPE. HR at 30 W demonstrated an additional significant interaction effect. However, the post-hoc comparisons confirmed that HR increased significantly from pre- to post-Ex in both conditions, but there were no significant differences between Pla and Caf at either time point. Although the estimation error changed over time (mean difference: −0.11 L·min⁻¹ in the Pla condition and − 0.18 L·min⁻¹ in the Caf condition), the magnitude of change did not differ between conditions (Fig. 2). Equivalence testing using the TOST procedure revealed that in the pre-Ex Caf and Pla condition, the lower bound test was significant, t(27) = 2.82, *p* < 0.01, but the upper bound test was not significant, t(27) = -0.85, *p* = 0.20. When testing the differences in estimation error between post-Ex Caf and Pla conditions, the lower bound test was not significant (t(27) = 1.54, *p* = 0.067), while the upper bound test was significant (t(27) = -2.16, *p* = 0.02). Together, these results indicate that the observed differences in estimation errors fell outside the predefined equivalence bounds of ± 0.1 L·min⁻¹. The estimation errors were not affected by body mass or cardiorespiratory fitness (measured VO_2_max). After adjustment for these covariates, there were no significant main effects of condition (*F*(1, 25) = 0.001, *p* = 0.981) or time (*F*(1, 25) = 0.12, *p* = 0.733), and no significant interaction effect (*F*(1, 25) = 0.22, *p* = 0.645). Neither body mass nor measured VO₂max had a significant moderating influence on the effects of condition or time (all *p* > 0.14).

**Table 3:** Values from the submaximal Ekblom-Bak cycle tests

	Pla pre-Ex	Pla post-Ex	Caf pre-Ex	Caf post-Ex	Condition F (df), *p =*	Time F (df), *p =*	Interaction F (df), *p =*
HR 30 W (bpm)	81 ± 15	103 ± 16	78 ± 12	104 ± 15	F(1,27) = 1.23, *p* = 0.28	F(1,27) = 275.82, *p <* 0.001	F(1,27) = 7.60, *p =* 0.01
					Partial η² = 0.04	Partial η² = 0.91	Partial η² = 0.22
HR high (bpm)	124 ± 15	140 ± 16	121 ± 13	140 ± 16	F(1,27) = 1.61, *p* = 0.22	F(1,27) = 181.62, *p <* 0.001	F(1,27) = 3.30, *p =* 0.08
					Partial η² = 0.06	Partial η² = 0.87	Partial η² = 0.11
RPE 30 W	7.4 ± 1.3	7.5 ± 1.3	7.1 ± 1.1	7.2 ± 1.4	F(1,27) = 0.280, *p* = 0.60	F(1,27) = 6.24, *p* = 0.02	F(1,27) = 2.43, *p* = 0.13
					Partial η² = 0.01	Partial η² = 0.19	Partial η² = 0.08
RPE high	13.5 ± 1.6	14.0 ± 1.8	13.1 ± 1.4	14.1 ± 1.2	F(1,27) = 0.911, *p* = 0.35	F(1,27) = 43.95, p = *p <* 0.001	F(1,27) = 2.39, *p* = 0.13
					Partial η² = 0.03	Partial η² = 0.62	Partial η² = 0.08
Estimated VO_2_max (L min^–1^)	3.64 ± 0.90	3.53 ± 0.90	3.69 ± 0.96	3.51 ± 0.91	F(1,27) = 0.273, *p* = 0.61	F(1,27) = 19.36, *p <* 0.001	F(1,27) = 1.96, *p =* 0.17
					Partial η² = 0.01	Partial η² = 0.42	Partial η² = 0.07
Estimation error (L min^–1^)^†^	– 0.17 ± 0.36	– 0.28 ± 0.35	– 0.12 ± 0.32	– 0.30 ± 0.33	F(1,27) = 0.15, *p* = 0.70	F(1,27) = 19.36, *p <* 0.001	F(1,27) = 1.96, *p =* 0.17
					Partial η² = 0.01	Partial η² = 0.42	Partial η² =0.07

Descriptive values are mean and standard deviation (SD)

*Pla* placebo, *Caf* caffeine, *Ex* exercise, *HR* heart rate, *bpm* beats per minute, *RPE* ratings of perceived exertion, *VO*_*2*_*max* maximal oxygen uptake

^†^The estimation error is the differences between the estimated and measured VO_2_max (i.e., estimated–measured VO_2_max)

## Discussion

This study investigated the effects of moderate caffeine intake (~ 3.3 mg·kg⁻¹) and prior strenuous exercise on HR responses and estimated VO₂max from the Ekblom-Bak (EB) test. Caffeine ingestion did not significantly affect HR or estimated VO₂max compared with placebo. No significant interaction was observed between caffeine and exercise conditions, indicating that caffeine did not influence the HR changes induced by prior physical exertion. In contrast, a pronounced time effect was evident, with higher HR and greater underestimation of VO₂max observed in the post-Ex condition compared with pre-Ex condition, regardless of treatment. In the post-Ex condition, the estimation errors were equivalent regardless of caffeine intake, and no differences were observed between the values. In the pre-Ex condition, there was no statistically significant difference; however, equivalence was not demonstrated, indicating that the effect of caffeine may be greater in the rested state. Estimation errors were not related to body mass or cardiorespiratory fitness. These findings indicate that moderate caffeine intake does not affect the validity of the EB-test, whereas performing the test after strenuous exercise leads to systematic underestimation of VO₂max due to elevated HR responses.

Test standardisations are important to produce valid and reliable VO_2_max estimations from submaximal exercise tests. Common preparations include refraining from smoking and vigorous physical activity the day before and on the test day, and not consuming a heavy meal within three hours prior to testing (Björkman et al. 2016; Ekblom-Bak et al. 2014). In clinical use, restrictions on caffeine intake are sometimes, but not always, part of the test preparations. The rationale for examining caffeine was based on the common assumption that it elevates HR and should therefore be avoided before HR-based submaximal tests. Another aspect of standardisation is that the individual should be in a rested state prior to testing. Both conditions were examined in the present study. The results are interpreted in consideration of previous research, and the possible physiological explanations and methodological implications are discussed below.

### Effects of caffeine on HR, estimated and measured VO₂max

Moderate caffeine ingestion did not significantly alter HR or VO₂max estimates, aligning with studies reporting minimal or no HR changes at submaximal intensities (Desbrow et al. 2012; Glaister and Gissane 2018). This is physiologically plausible given that moderate caffeine doses primarily exert their effects through adenosine receptor antagonism and mild sympathetic stimulation (Spriet 2014), which in trained individuals may be counterbalanced by high resting vagal tone and an enhanced capacity to maintain stroke volume during incremental work (Boutcher et al. 2003; Rowland 2009; Sampaio-Jorge et al. 2021). Consequently, the magnitude of the estimation errors observed following caffeine intake (-0.12 L·min⁻¹) was broadly consistent with previous reports of estimation errors around − 0.05 L·min⁻¹ in age-matched individuals (Björkman et al. 2016). This might partly be explained by the use of the variable ΔHR (defined as the difference in HR between the high and low work rates) in the EB-test prediction equation (Ekblom-Bak et al. 2014). The ΔHR variable reduces the impact of variability in absolute submaximal HR responses due to factors as ambient temperature, nervousness, and more. Therefore, it may be of lesser importance to follow certain standardisation procedures, intended to reduce the influence of the sympathetic nervous system on the submaximal HR response in a single-point test (Astrand and Ryhming 1954; Brouha 1943; Ebbeling et al. 1991; Ryhming 1953). However, if an external factor exerts a greater effect on, for example, the low work rate, this will shift the slope of the HR response curve and lead to greater estimation error.

The use of caffeine abstention prior to submaximal testing is a practice likely influenced by the inconsistent evidence surrounding caffeine’s effects during submaximal exercise (Bangsbo et al. 1992; Bell et al. 1998; Daniels et al. 1998; Desbrow et al. 2012; Engels et al. 1999; Flinn et al. 1990; Gaesser and Rich 1985; Glaister et al. 2021, 2025; Laurence et al. 2012; McClaran and Wetter 2007; Tarnopolsky et al. 1989; Turley and Gerst 2006) and its well-established ergogenic benefits (Chen et al. 2024; Yu and Ding 2025). Furthermore, the results from previous studies are not directly applicable to the EB-test work rates, as the 30 W load represents a lower exercise intensity than what is commonly studied. Nevertheless, the present findings among healthy, well-trained, habitual caffeine consumers do not support the use of caffeine abstention as a standardisation criterion for the EB test. However, individuals with conditions such as hypertension, who may have impaired blood pressure or blood flow regulation, or those who are non-habitual caffeine consumers, could respond differently to caffeine ingestion. In such populations, abstaining from caffeine prior to testing may still be advisable to minimise variability in cardiovascular responses.

The inconsistencies observed across studies examining caffeine’s effects on HR during submaximal exercise likely stem from differences in dose, timing, habitual caffeine intake, exercise intensity, and participant fitness level. For instance, non-habitual caffeine consumers have shown reduced HR at low and moderate intensities after ingesting small doses (1.5–3 mg·kg⁻¹) compared with placebo (McClaran and Wetter 2007), suggesting an attenuated cardiovascular response to caffeine at these levels. Similar reductions in HR have also been observed in endurance-trained individuals with moderate habitual caffeine use (Glaister et al. 2021). In the present study, no significant differences were found in HR or VO₂max estimates following moderate caffeine ingestion in habitual caffeine users. In contrast, studies employing higher doses (≥ 6 mg·kg⁻¹) or more demanding protocols, such as time trials, have reported increased HR following caffeine ingestion (Desbrow et al. 2012; Laurence et al. 2012).

The caffeine dose in the present study is equivalent to approximately two large cups of strong coffee or one and a half 500-ml cans of energy drink, resulting in a relative intake just above 3 mg·kg⁻¹ body mass among the participants. The dose used (3–4 mg·kg⁻¹) was moderate and falls within the range of caffeine intake shown to produce optimal ergogenic effects (Guest et al. 2021). Caffeine was ingested 50 min prior to the start of the first EB-test, which falls within the time window for peak performance benefits (Guest et al. 2021). However, it is worth noticing that the second EB-test was completed approximately two hours after caffeine intake, when peak plasma concentrations are expected to be attenuated. The absence of HR changes despite a moderate rise in BP further supports effective caffeine uptake without major systemic cardiovascular stimulation. While measured VO₂max and maximal HR were unchanged by caffeine, time to exhaustion increased, confirming an ergogenic effect on endurance performance.

### Effects of prior exercise on HR and estimated VO₂max

Strenuous exercise prior to testing increased HR during the EB-test, compromising test validity by causing a significantly higher systematic underestimation of VO₂max compared to tests in the rested state. The significantly elevated HR across work rates (22–26 bpm during the 30 W work rate and 16–19 bpm during the higher work rate, respectively) likely reflects residual sympathetic activity and post-exercise cardiovascular adjustments. Factors such as elevated core temperature, circulating catecholamines, and incomplete cardiovascular recovery prolong autonomic activation, delaying the return of HR to baseline (Romero et al. 2017). These exercise-induced disruptions alter the linear HR–work rate relationship assumed by submaximal tests, reducing the accuracy of VO₂max predictions. Furthermore, recovery is influenced by individual characteristics: highly trained individuals generally experience faster HR recovery (Darr et al. 1988) whereas vagal reactivation is impaired in populations with metabolic syndrome (Sung et al. 2006). In the present study, a 30-minute rest period separated the strenuous exercise from the second EB-test. However, there is a lack of studies systematically investigating the time required for full circulatory recovery. It is likely that 30 min of rest after a very intense exercise session is insufficient for HR and other cardiovascular variables to return to baseline, even if the participant subjectively feels recovered. Whether one or two hours, or a recovery period integrated into a training session, would be sufficient remains unclear. The results from the present study highlight the necessity of allowing sufficient recovery time following intense exercise to ensure cardiovascular variables have returned closer to baseline before conducting HR–based fitness assessments.

### Influence of body mass and fitness level

Body mass and cardiorespiratory fitness did not significantly influence estimation accuracy, indicating that the EB-test remains robust across a range of fitness levels and body sizes. This finding aligns with previous validation studies (Björkman et al. 2016), which have demonstrated consistent predictive validity across demographic subgroups.

### Limitations

The study’s strengths include its randomized, double-blind, placebo-controlled, crossover design and the use of a validated submaximal test. The caffeine dose was moderate and ecologically relevant, approximating the amount in two large cups of strong coffee. Participants abstained from caffeine for at least 24 h before testing, ensuring acute withdrawal and consistent baseline conditions. Although the study was conducted using a double-blind design, 12 of the 28 included participants spontaneously reported that they believed they were in the caffeine condition. Importantly, participants were not asked to guess or identify the condition at any point during the study; these reports were entirely voluntary and unprompted and were noted by the researchers when they occurred. All such reports occurred during caffeine sessions and were correct, suggesting that complete blinding was not fully maintained. This may be attributed to the recognisable physiological and psychological effects of caffeine, such as increased alertness, which are difficult to mask even in well-controlled, double-blind protocols. The partial unblinding could have influenced participants’ perceptions and performance through expectancy effects, where individuals anticipate improved performance following caffeine ingestion. Consequently, some of the observed effects may reflect psychological rather than purely physiological responses. Interestingly, it has been demonstrated that the effect on perceived exertion is modulated by individual expectations of ergogenic effects (Azevedo et al. 2021).

Examining the effects of caffeine on hemodynamic responses presents significant methodological challenges. The observed outcomes are markedly influenced by the administered dose, the timing of ingestion, and the route of administration. In the present study, only one caffeine dose and one type of submaximal test were used; therefore, results may differ when using other doses, populations, or testing protocols. Finally, the sample size in the present study is relatively small. The initial power calculation indicated that 37 participants would be required to detect the targeted effect with 80% power, and only 28 participants were ultimately enrolled due to recruitment challenges and unexpected dropouts. This means the study was underpowered relative to the original design, and some small physiological changes may have gone undetected. Although no significant differences in estimation errors were observed between conditions, equivalence testing indicated that these differences could not be confidently considered negligible within the predefined bounds (± 0.1 L·min⁻¹). This is likely related, at least in part, to the limited sample size. Consequently, small caffeine-induced changes in estimation error cannot be completely ruled out, and future studies with larger samples are needed to confirm these findings. Nevertheless, the actual and observed effects are unlikely to reflect Type II errors, as the measured changes are genuine findings rather than statistical artefacts. Therefore, while underpowering may limit the ability to detect subtle effects, the main results presented remain robust and meaningful.

Future research should examine the effects of caffeine and prior exercise on the validity of other submaximal tests across different populations (e.g., older adults, less-trained individuals) and a range of caffeine doses. Studies could also explore the optimal duration of rest before submaximal testing (e.g., 2 h, 12 h, or one day) to determine the recovery period required for accurate VO₂max estimation from HR-based tests. The present study was conducted in healthy, physically active individuals, using a single caffeine dose and a single type of submaximal exercise test. Additionally, the strenuous prior exercise followed by a short recovery period represents an extreme scenario that may not accurately reflect most real-world situations (i.e., typical daily activity or athletic routines). Untrained individuals can often perform a maximal test, but their VO_2_max are lower, and following intense exercise, they would likely exhibit even higher HR responses. Thus, while the general patterns observed here may extend to less trained populations, the magnitude and timing of cardiovascular responses may differ. Consequently, caution is warranted when extrapolating these results to broader populations or more ecologically representative conditions. Nevertheless, until further evidence is available, it remains prudent to follow the standard recommendation of avoiding strenuous or vigorous activity the day before or on the test day of submaximal exercise assessments.

## Conclusion

Moderate caffeine intake (~ 3–4 mg·kg⁻¹) did not compromise the accuracy of VO₂max estimation using the EB-test, indicating that abstaining from usual caffeine intake may be unnecessary for healthy habitual consumers prior to testing. In contrast, performing the test after strenuous exercise elevated submaximal HR and led to systematic underestimation of VO₂max, reflecting residual sympathetic activation and incomplete recovery. These results indicate that while the EB-test is robust to moderate caffeine ingestion, it is sensitive to pre-test physical load. Practically, submaximal HR-based tests should therefore be performed in a rested state to ensure valid and reliable VO₂max estimation.

## Data Availability

Data are available from the corresponding author upon reasonable request.

## Abbreviations

ANCOVA: Analysis of covariance
ANOVA: Analysis of variance
BP: Blood pressure
Bpm: Beats per minute
Caf: Caffeine
EB-test: Ekblom-Bak test
HR: Heart rate
L min^− 1^: Litres per minute
mg·kg⁻¹: Milligrams per kilogram
Pla: Placebo
RM: Repeated measures
RPE: Rating of perceived exertion
SD: Standard deviation
SE: Standard error
VO_2_max: Maximal oxygen uptake
